# Association of 25 bp Deletion in *MYBPC3* Gene with Left Ventricle Dysfunction in Coronary Artery Disease Patients

**DOI:** 10.1371/journal.pone.0024123

**Published:** 2011-09-07

**Authors:** Anshika Srivastava, Naveen Garg, Tulika Mittal, Roopali Khanna, Shipra Gupta, Prahlad Kishore Seth, Balraj Mittal

**Affiliations:** 1 Department of Genetics, Sanjay Gandhi Post Graduate Institute of Medical Sciences Lucknow (UP), Lucknow, India; 2 Department of Cardiology, Sanjay Gandhi Post Graduate Institute of Medical Sciences Lucknow (UP), Lucknow, India; 3 Bioinformatic Center, Biotech Park Lucknow (UP), Lucknow, India; Universite de Montreal, Canada

## Abstract

**Rationale:**

Mutations in *MYBPC3* encoding cardiac myosin binding protein C are common genetic cause of hereditary cardiac myopathies. An intronic 25-bp deletion in *MYBPC3* at 3′ region is associated with dilated (DCM) and hypertrophic (HCM) cardiomyopathies in Southeast Asia. However, the frequency of *MYBPC3* 25 bp deletion and associated clinical presentation has not been established in an unrelated cohort of left ventricular dysfunction (LVD) secondary to coronary artery disease (CAD) patients.

**Objective:**

We sought to determine the role of *MYBPC3* 25 bp polymorphism on LVD in two cohorts of CAD patients.

**Methods and Results:**

The study included 265 consecutive patients with angiographically confirmed CAD and 220 controls. *MYBPC3* 25 bp polymorphism was determined by polymerase chain reaction. Our results showed that carrier status of *MYBPC3* 25 bp deletion was associated with significant compromised left ventricle ejection fraction (LVEF ≤45) in CAD patients (p value  =  <0**.**001; OR = 4.49). To validate our results, we performed a replication study in additional 140 cases with similar clinical characteristics and results again confirmed consistent findings (p = 0.029; OR = 3.3). Also, presence of the gene deletion did not have significant association in CAD patients with preserved ejection fraction (LVEF>45) (p value  = 0.1; OR  = 2.3).

**Conclusion:**

The frequency of *MYBPC3* DW genotype and D allele was associated with compromised LVEF implying that genetic variants of *MYBPC3* encoding mutant structural sarcomere protein could increase susceptibility to left ventricular dysfunction. Therefore, 25 bp deletion in *MYBPC3* may represent a genetic marker for cardiac failure in CAD patients from Southeast Asia.

## Introduction

Coronary artery disease (CAD) is defined as atherosclerotic blockage[1] of arteries, supplying oxygen rich blood to the myocardium (heart muscle), is the most common cause of fatality, disability and economic loss, particularly in industrialized countries. While the signs and symptoms of coronary artery disease are noted in advanced state of disease, most individuals remain asymptomatic for decades. Furthermore, major complication that some CAD patients face over time is the development of Left Ventricle dysfunction (LVD). The LVD usually leads to heart failure, arrhythmias and other cardiovascular complications. Besides poorly developed collaterals, extensive atherosclerotic disease and large myocardial infarction are traditional predictors of LVD. However, in clinical practice, it is observed that some CAD patients who do not have such complications but still develop LVD, whereas others with well-defined predictors do not develop LV dysfunction, which is quite puzzling.

Earlier genetic studies have correlated LVD with dilated cardiomyopathy (DCM).[2] Mutations in several genes including those encoding sarcomeric proteins such as myosin heavy polypeptide (MYH7), cardiac troponin I (*TNNI3*),[3] cardiac myosin binding protein C (*MYBPC3*)[4] have been identified in DCM.[5], [6], [7]

Myosin-binding protein C (MyBP-C) is a key constituent of cardiac muscle thick filaments, which by interacting with myosin,[8], [9], [10] titin[11] and actin[12] contributes to the structural integrity of the sarcomere and regulates cardiac contractility in response to adrenergic stimulation.[13], [14] Mutations in *MYBPC3* result in disorganized sarcomeric structure, thus *MYBPC3* has emerged as an important gene for increased risk of heart failure through cardiomyopathies (DCM and HCM).[15], [16], [17], [18]

A common polymorphic intronic deletion of 25-base- pair in *MYBPC3* at the 3′ region of the gene [19] has been recently reported to be associated with DCM and HCM in populations of Southeast Asia.[20] The deletion in intron 32 causes skipping of the downstream exon 33 and results in incorporation of mis-sense amino acids at C-terminal region of the protein.[19], [20] The 25-bp deletion is present in 2–6% of individuals in Indian populations and high incidence of cardiac diseases has been partly attributed to the existence of this polymorphism. Two community based studies carried out in UK reported significant ethnic differences in left ventricular systolic function and underlying CAD between South Asians and European white subjects.[21], [22] Moreover, a close relation between LV ejection fraction (LVEF) and mortality has also been demonstrated. Therefore, we carried out the present study to assess if the common polymorphism in *MYBPC3* has any role in LV dysfunction. To the best of our knowledge, it is the first study to report the association of 25 bp polymorphism of *MYBPC3* with low left ventricular ejection fraction in CAD patients.

## Results

### 1.1 Patient characteristics

Demographic and lipid profile of healthy subjects is shown in Table 1. Clinical characteristics of CAD patients -Primary, replication and combined cohorts of study are shown in Table 2. In combined cohort, there was no significant difference in the mean age of CAD patients and controls. The male/female ratio was comparable in both CAD cases as well as in controls. Evaluation of the defined risk factors in the cohort showed that 45.9% patients were hypertensive and 28.4% patients were diabetic. Moreover, 26.4% patients were associated with smoking. Patients with stable angina were 31.4% and unstable angina/ Non ST Segment Elevation Myocardial Infarction (NSTEMI) formed 20.5% of the clinical syndrome. ST Segment Elevation Myocardial Infarction (STEMI) patients with anterior wall myocardial infarction (AWMI) and inferior wall myocardial infarction (IWMI) were 28.1% and 19.5% respectively. Only two patients were found to be affected with lateral wall myocardial infarction (LWMI) (0.5%).The angiographic profile categorized patients with single vessel disease (SVD), double vessel disease (DVD), and triple vessel disease (TVD) as 68.9%, 24.7% and 6.4% respectively. The mean ejection fraction was 50.0±11.18. The kurtosis and skewness for left ventricular ejection fraction (LVEF) were 0.12 and −0.98. Thus, the data appeared to be normally distributed. We considered ≤45% LVEF cut off as significant LVD. Of the total 405 CAD patients, 74.1% showed preserved (>45%) ejection fraction while 25.9% had compromised ejection fractions (≤45%) and were categorized as having significant LVD. All the values in primary stage matched to replication stage and there was no significant difference in the values of three stages of the study.

**Table 1:** Demographic and lipid profile of healthy subjects.

Number (n) = 220	
Age- yr±SD	54.94±8.12
Male sex n (%)	173 (78%)
Lipid Levels	
a) High Density Lipoprotein (mg/dl)	34.29±2.62
b) Low Density Lipoprotein (mg/dl)	110.00±8.68
c) Triglycerides (mg/dl)	154.46±10.04
d) Total cholesterol (mg/dl)	163.26±8.55

**Table 2:** Clinical Characteristics of CAD patients.

CLINICAL CHARACTERISTICS	Primary Stage	Replication Stage	Combined
Total Subjects	**265**	**140**	**405**
*Age – yr	56.52±10.20	54.36±8.179	55.78±9.59
Male sex	225 (84.9)	123 (87.9)	348 (85.9)
**RISK FACTORS**			
Hypertension	119 (44.9)	67 (47.9)	186 (45.9)
Diabetes	77 (29.1)	38 (27.1)	115 (28.4)
Smoking	67 (25.3)	40 (28.6)	107 (26.4)
*Lipid Levels			
a) High Density Lipoprotein (mg/dl)	32.31±7.91	31.88±5.86	32.19±7.45
b) Low Density Lipoprotein (mg/dl)	100.13±24.33	103.74±22.90	100.97±24.40
c) Triglycerides (mg/dl)	155.64±69.19	125.41±35.54	149.11±62.25
d)Total cholesterol (mg/dl)	170.30±21.98	141.75±39.61	163.74±30.53
**CLINICAL SYNDROME**			
Stable angina	82 (30.9)	45 (32.1)	127 (31.4)
Unstable angina/Non ST Elevation Myocardial Infarction (NSTEMI)	59 (22.3)	24 (17.1)	83 (20.5)
ST Elevation Myocardial Infarction (STEMI)	124 (46.8)	71 (50.7)	195 (48.1)
Anterior wall myocardial infarction (AWMI)	72 (27.2)	42 (30)	114 (28.1)
Inferior wall myocardial infarction (IWMI)	51 (19.2)	28 (20)	79 (19.5)
Lateral wall myocardial infarction (LWMI)	1 (0.4)	1(0.7)	2 (0.5)
**ANGIOGRAPHIC PROFILE**			
Single vessel disease (SVD)	165 (62.3)	114 (81.4)	279 (68.9)
Double vessel disease (DVD)	77 (29.1)	23 (16.4)	100 (24.7)
Triple vessel disease (TVD)	23 (8.7)	3 (2.1)	26 (6.4)
**LEFT VENTRICULAR FUNCTION**			
*Mean Left Ventricle Ejection Fraction (LVEF)	50.70±11.65	48.56±10.2	50.0±11.18
>45	206 (77.7)	94 (67.1)	300 (74.1)
≤45	59 (22.3)	46 (32.9)	105 (25.9)

*Values are mean ± SD.

### 1.2 Influence of MYBPC3 25 bp Deletion on CAD and its clinical characteristics

Distributions of genotypes of *MYBPC3* gene deletion in controls were in accordance with Hardy-Weinberg equilibrium (p = 0.98). Considering the low allelic frequency allele, we also applied fisher exact test for conditional probability of heterozygous variants which was found to be p = 0.97.

After this we compared the genotype and allele frequency of *MYBPC3* 25 bp polymorphism between CAD patients (both stages) and healthy controls. Our results suggested significant association of MYBPC3 gene deletion with CAD [Table 3 (Primary; Replication); DW genotype p value  = 0.03; <0.01: D allele p value  = 0.03; <0.01]. The frequency of *MYBPC3* gene deletion in total (Primary and replication cohorts) CAD patients was also significantly higher (p value = 0.003; Table 4) than healthy controls. As there were no homozygous DD genotyped individuals in both cases and controls, the heterozygous carrier (DW) itself associated with CAD.

**Table 3:** Distributions for 25 bp deletion in MYBPC3 in CAD patients and Healthy controls.

Genotypes/Allele	HC (220)N (%)	CAD^a^(265)N (%)	CAD^b^(140)N (%)	p– value^a^OR (95% CI)	p– value^ b^OR (95% CI)
WW	215 (97.7)	248 (93.6)	123 (87.9)	1(reference)	1(reference)
DW+DD	5 (2.3)	17 (6.4)	17 (12.1)	**0.03 ; 3.0 (1.1**–**8.5)**	**<0.01 ; 6.8 (2.3**–**19.3)**
W	435 (98.9)	513 (96.8)	263 (93.9)	1(reference)	1(reference)
D	5 (1.1)	17 (3.2)	17 (6.1)	**0.03 ;** **3.0 (1.0**–**8.2)**	**<0.01 ; 6.3 (2.2**–**17.5)**

CAD-Coronary artery disease, HC-Healthy control, OR–Odds Ratio, CI–Confidence interval.

D, allele with deletion; W, wild-type allele;

a CAD patients in Primary cohort; ^b^CAD patients in Replication cohort.

a =  represents the p value for the comparison of carriers (D,W + D,D) and non-carriers (W,W) in CAD patients (primary cohort) and HC.

b =  represents the p value for the comparison of carriers (D,W + D,D) and non-carriers (W,W) in CAD patients (replication cohort) and HC.

Significant values are shown in BOLD.

**Table 4:** Overall genotypic and allelic frequency of *MYBPC3* in total CAD patients.

Genotypes/ Allele	HC (220)N (%)	CAD(405)N (%)	p– valueOR (95% CI)
WW	215 (97.7)	371 (91.6)	1(reference)
DW	5 (2.3)	34 (8.4)	**0.003 ; 4.3 (1.6**–**11.2)**
W	435 (98.9)	776 (95.8)	1(reference)
D	5 (1.1)	34 (4.2)	**0.003 ; 4.1 (1.5**–**10.6)**

Significant values are shown in BOLD.

Further, we compared the distribution of wild type (WW) genotype of *MYBPC3* (no deletion) and DW (Carrier of one deleted allele) with clinical characteristics of CAD. There were no significant differences between frequencies of WW and DW with reference to clinical characteristics of CAD, except with compromised LVEF (Table 5. 23.5% vs. 52.9%, p- value = <0.01) Also, a borderline significance (p value <0.05) was also observed in case of triple vessel disease. When CAD patients were divided on the basis of associated phenotypes like diabetes mellitus, hypertension or risk factors like smoking status of the subjects carrying wild type and deleted allele, the *MYBPC3* gene deletion did not modulate the risk of CAD due to these phenotypes or factors.

**Table 5:** Effect of MYBPC3 deletion on clinical characteristics in total CAD patients.

Variable	MYBPC3 (WW)N = 371 (%)	MYBPC3 (DW)N = 34 (%)	P value
Stable Angina	113 (30.5)	14 (41.2)	NS
Unstable Angina	54 (14.6)	3 (8.8)	NS
Anterior wall Myocardial Infarction	103 (27.8)	9 (26.5)	NS
Inferior wall Myocardial Infarction	71 (19.1)	8 (23.5)	NS
Single Vessel Disease (SVD)	257 (69.3)	22 (64.7)	NS
Double Vessel Disease (DVD)	93 (25.1)	7 (20.6)	NS
Triple Vessel Disease (TVD)	21 (5.7)	5 (14.7)	**<0.05**
LVEF <45	87 (23.5)	18 (52.9)	**<0.01**

Significant values are shown in BOLD; NS =  Non-significant.

### 1.3 Influence of MYBPC3 25 bp Deletion on CAD patients with reduced and preserved ejection fraction

In case-only analysis, first we applied linear model on all CAD patients and found 25 bp gene deletion to be significantly associated with compromised LVEF (p value  =  <0.002 [B = 6.3] Table 6). Then we segregated CAD patients on the basis of compromised (</ = 45%) and preserved (>45%) left ventricular ejection fraction and compared with their status of *MYBPC3* gene polymorphism. We found that higher percentage of CAD patients carrying heterogeneous DW genotype had compromised ejection fraction as compared to the patients with preserved ejection fraction. This difference was statistically significant (p value  = 0.004 OR  = 4.32 (1.58 – 11.82) Table 7). In addition, we performed a replication study on 140 more CAD patients and compared with primary cohort. The effect was consistently stronger in the CAD patients with reduced ejection fraction than CAD patients with preserved ejection fraction subgroups (p value  = 0.029 OR = 6.208 (3.3 (1.1–10.1)] Furthermore, we calculated the z and p values to look into the overall significance and the results again confirmed the association of 25 bp gene deletion with compromised ejection fraction (Table 7).

**Table 6:** Parameter Estimate Applying General Linear Model.

		All Subjects	WW	DW+DD	P-value
**No of Subjects**		405	371	34	
	LVEF	50.0±11.1	50.5±10.92	44.26±12.5	**<0.002**

Significant value is shown in BOLD.

**Table 7:** Distributions of 25 bp deletion in *MYBPC3* in CAD patients with preserved (LVEF >45) and compromised (LVEF ≤45) Left Ventricular ejection fraction.

Primary Stage				
LVEF Categorical	Genotypes	Total CADN (%)	p– value	OR (95% CI)
>45	WW	198 (96.1)	**-**	**1 (reference)**
	DW+DD	8 (3.9)		
≤45	WW	50 (84.7)	**<0.001**	**4.32 (1.58**–**11.82)**
	DW+DD	9 (15.3)		

Replication stage				
LVEF Categorical	Genotypes	Total CADN (%)	p– value	OR (95% CI)
>45	WW	86 (91.5)	**-**	**1 (reference)**
	DW+DD	8 (8.5)		
≤45	WW	37 (80.4)	**0.029**	**3.3 (1.1**–**10.1)**
	DW+DD	9 (19.6)		

*Z overall **3.218***.

*P value **<0.001***.

Significant values are shown in BOLD.

We looked for the association of *MYBPC3* gene deletion on LVD by changing the cut off values for LVEF to <40% and <50% also. The level of significance decreased when we considered the cut off for LV dysfunction to 50% and at 40% cut off, level of significance increased but we would have missed some patients with LV dysfunction. Therefore, we selected the cut off to 45% to include definite patients with LV dysfunction.

### 1.4 Frequency Distribution of MYBPC3 25 bp deletion polymorphism in CAD patients with preserved and compromised LVEF and Controls

To further evaluate whether *MYBPC3* gene deletion is associated with CAD or LVEF, we analyzed the status of gene deletion in CAD patients with preserved and compromised LVEF. Of the total 405 CAD patients, 105 CAD patients had ejection fraction ≤45 while remaining 300 had preserved LVEF (>45). On comparing the genotype frequency distribution of these two groups of patients with that of healthy controls it was observed that only carriers of DW genotype were associated with LVEF [DW genotype p value  =  <0.001 OR  = 11.6 (3.9–34.2)]; D allele p value  =  <0.001 [9.8 (3.5–27.6)] while no risk of DW genotype was observed in CAD patients with preserved ejection fraction [Table 8; DW genotype p value  = 0.09; OR = 2.3 (0.8–6.6); D allele (p = 0.1;OR = 2.3 (0.8–6.4)] between these two groups. It suggests that *MYBPC3* 25 bp deletion does not show any association with CAD patients having preserved LVEF.

**Table 8:** Frequency Distribution of MYBPC3 25 bp deletion polymorphism in CAD patients with preserved and reduced LVEF and Controls.

Genotypes/ Allele	HC (220) N (%)	CAD with preserved LVEF (300) N (%)	CAD with reduced LVEF (105) N (%)	p–value & OR (95% CI) for CAD with preserved LVEF^a^	p– value & OR (95% CI) for CAD with reduced LVEF^b^
WW	215 (97.7)	284 (94.7)	87 (82.9)	1(reference)	1(reference)
DW/DD	5 (2.3)	16 (5.3)	18 (17.1)	0.09 [2.3 (0.8–6.6)]	**<0.001 [11.6 (3.9**–**34.2)]**
W	435 (98.9)	584 (97.3)	192 (91.4)	1(reference)	1(reference)
D	5 (1.1)	16 (2.7)	18 (8.6)	0.1 [2.3 (0.8–6.4)]	**<0.001 [9.8 (3.5**–**27.6)**

D: allele with deletion; W: wild-type allele. Numbers in parentheses indicate the proportion of each genotype/allele as a percentage of total cases or controls. OR, odds ratio for the likelihood CAD patients with preserved LVEF^a^ and reduced LVEF^b^ versus Healthy Controls (HC).

a =  represents the p value for the comparison of carriers (D,W + D,D) and non-carriers (W,W) in CAD patients with preserved LVEF and HC.

b =  represents the p value for the comparison of carriers (D,W + D,D) and non-carriers (W,W) in CAD patients with reduced LVEF and HC.

Generally, CAD patients with ST elevation MI are more prone to develop LV dysfunction which was also evident in the present study. Therefore, we looked for any significant interaction of STEMI with MYBPC3 deletion status and low Ejection fraction but found borderline significance (p value = 0.04) whereas in case of CAD patients with low LVEF and no history of STEMI, the association with MYBPC3 gene deletion was highly significant (p value = 0.001). It suggests that MYBPC3 gene deletion is influencing development of LVD in CAD patients without history of STEMI.

## Discussion

The main finding of the present study suggests close relationship between the *MYBPC3* 25 bp polymorphic deletion, a common *MYBPC3* variant, and significantly higher risk of severe left ventricular dysfunction (LVD) in CAD patients.

Notably, LVD is a complex condition that emerges as a common pathway for a host of cardiac disorders. The LVD results from the changes in the structure and function of heart muscle as well as changes in collagen and other cardiac proteins.

Previously, a number of mutations in the genes for sarcomeric proteins have been found to be associated with the risk of DCM [7] and some of them are shown to cause sarcomeric disorganization, which is believed as one of the mechanisms by which pathogenesis is triggered in the heart.[8], [23] In addition to genetic predisposition, viral infection, molecular mimicry, and oxidative stress are potential contributing factors to dilated cardiomyopathy. These factors are believed to be present before DCM and lead to severe LV dysfunction. Individuals, who are genetically predisposed, when exposed to any of these contributory factors, may develop LVD.

Similarly, we believe that CAD patients carrying the 25 bp polymorphic deletion in *MYBPC3* when triggered by severe ischemic insult to the cardiac muscle cell (in place of viral infection or other contributory factors for the development of DCM) may be more likely to develop severe LV dysfunction in comparison to CAD patients who are not carrying this mutation.

A large number of studies during last ten years have clearly confirmed that cardiac myosin binding protein C plays pivotal role in the genesis of cardiac muscle disorders.[20] The 25 bp intronic deletion causes skipping of exon 33 and incorporation of mis-sense of amino acids at the C-terminal.[19], [20] The mutated protein has been shown to incorporate in the myofibrils [20] and may cause breakdown of sarcomere.

Several mechanisms have been proposed to explain pathogenesis of cardiac muscle due to truncated and mis-sense myosin binding protein C. These include poison peptides, haplo-insufficiency caused by nonsense-mediated mRNA decay and impairment of ubiquitin–proteasome system (UPS). The missense MYBPC3 mutations have been reported to destabilize its proteins through UPS and it may contribute to cardiac dysfunction through impairment of the ubiquitin-proteasome system.[24] It has been shown that mutant cMyBP-C protein is preferentially degraded by the ubiquitin proteasome system (UPS), which, in turn, may competitively inhibit breakdown of other UPS substrates. Also, taking into consideration the decline in function of the UPS with age and oxidative stress [25], [26] the altered protein may simply accumulate, disrupt the cellular homeostasis and initiate LVD

A recent study from UK explored any ethnicity-related differences in left ventricular function, structure and geometry in a population based study of UK Indian Asian and European Whites [22] and they observed significant differences in cardiac structure and sensitive parameters of LV function. Earlier, another UK based study did not observe differences in incidence of LVSD but reported that majority of patients originating from Indian subcontinent had CAD as underlying cause of LVSD.[21] As *MYBPC3* 25 bp deletion is mainly confined to Southeast Asia,[20] it is possible that some of the differences in two groups (Southeast Asians and Whites) might be associated with the common *MYBPC3* 25 bp deletion.

So far, the influence of 25 bp *MYBPC3* intronic deletion on LVD in patients with DCM was known.[27] However, our results show that CAD patients with *MYBPC3* 25 bp deletion also become more prone to LVD. Presently there are no such methods or tests available to pinpoint CAD patients who are at higher risk of developing severe cardiac disorder in later stages. The late onset symptoms and influence of secondary risk factors may cause a lasting threat to the carriers. However, the gene-based insights into pathophysiology may allow more subtle clinical manifestations to be identified and additional phenotypes associated with the variant to be investigated. Furthermore, genotyping could be used for the identification of persons at risk of severe LV dysfunction and heart failure among various Indian populations and migrants of Indian origin. Thus, could be accompanied by appropriate medications and advice for a lower-risk lifestyle.

### Study limitations

The sample size in our present study is limited, therefore it will require confirmation in larger cohorts. Because this is an association study, we cannot rule out the presence of possible linkage disequilibrium with other neighboring genes that might explain the significant association with atherosclerotic phenotype or adverse prognosis.

Moreover, it is a retrospective study, so it must also be carried out prospectively before clinical application. Notably, the study was conducted in patients with severe CAD undergoing angioplasty and it is not certain whether the patients had LVD before ischemic insult or the left ventricular dysfunction developed later.

### Conclusion

Our study suggests that *MYBPC3* 25 bp polymorphic deletion is associated in CAD patients with compromised left ventricular ejection fraction. It implies that a common genetic variant of *MYBPC3* encoding structural sarcomere protein could increase susceptibility to left ventricular dysfunction, particularly following an ischemic event. These findings add new evidence to existing data on the linkage between *MYBPC3* function and outcome in patients in later stages in the cardiac failure. Therefore, the *MYBPC3* 25 bp deletion may be explored as biomarker for the development of severe LV dysfunction in CAD patients of persons originating from Indian subcontinent.

## Materials and Methods

### Ethics Statement

The institutional ethical committee of Sanjay Gandhi Post Graduate Institute of Medical Sciences (SGPGIMS) approved the study protocol, and the authors followed the norms of World's Association Declaration of Helsinki. All the participants were provided with written informed consent for the study.

### 2.1 Study Population

The present study was carried out in two stages, primary and replication stages. In the primary stage, we studied 265 CAD patients recruited from July 2008 to December 2009. In the replication stage, further 140 cases were enrolled. The diagnostic parameters used in the primary stage were also applied to the replication stage

Both the primary and replication cohorts had significant coronary artery disease, (diagnosis, confirmed by coronary angiography and further all these subjects underwent coronary angioplasty) recruited from the Department of Cardiology of Sanjay Gandhi Postgraduate Institute of Medical Sciences (SGPGIMS), Lucknow, Uttar Pradesh, India. The detailed clinical history of CAD patients was based on hospital investigations including angiography. Angiographically identified stenoses >70% in the major coronary vessels at the time of the study were used to classify patients as having single-vessel, double-vessel, or triple-vessel disease.

The control (non-CAD) population consisted of 220 subjects (173 males and 47 females) (mean age years 54.94±8.12.) with no clinical evidence of CAD or LV dysfunction (by echocardiography) and also without positive family history of CAD or myocardial infarction (MI). In addition, the inclusion criteria for controls were absence of prior history of high systolic blood pressure, abnormal lipid profile, diabetes mellitus and obesity. Both patient and control were frequency-matched to age, gender and ethnicity. After obtaining informed consent, all the individuals were personally interviewed for information on food habits, occupation and tobacco usage.

### 2.2 Data collection

The clinical data was obtained by reviewing the patient's medical records. Left ventricle ejection fraction (LVEF) was calculated quantitatively by echocardiography, just before angiography procedure, using the Simpson's method.[28] Echocardiography was repeated in 10% of patients and results were totally concordant. Hypertension was defined as systolic blood pressure >140 mmHg or a diastolic blood pressure >90 mmHg or patients using antihypertensive drugs. Smoking was classified as smokers (ex-smoker and current smokers) and non smokers. Similarly, diabetes mellitus was defined as patients with fasting plasma glucose >6.9 mmol/L or patients using anti-diabetic medication. All laboratory parameters, as stated in the medical record, were determined in fasting patients. Total cholesterol, high-density lipoprotein (HDL) cholesterol and triglyceride levels were measured by standard enzymatic methods. LDL cholesterol concentrations were calculated using the Friedewald's formula.[29]

### 2.3 Genetic Analysis

Genomic DNA was isolated from peripheral blood leukocytes according to a standard salting out method. [30] The polymorphism was genotyped using the polymerase chain reaction method. As a negative control, PCR mix without DNA sample was used to ensure contamination free PCR product. Laboratory personnel were blinded to the case–control status of the subjects and genotyping was done using a pair of primers [20] flanking the 25 bp deletion region in *MYBPC3.* PCR products (Wild Type [WW]: 403 bp and mutant [DW]: 378 bp) were analysed on 6% polyacrylamide gel. Ten percent of samples from patients and controls were sequenced to evaluate the quality of genotyping, which showed 100% concordance.

### 2.4 Statistical analysis

Descriptive statistics were presented as mean and standard deviation (SD) for continuous measures while absolute value and percentages were used for categorical measures. The chi-square goodness of fit test was used for any deviation from Hardy Weinberg Equilibrium in controls and fisher exact test was applied as given by Emigh [31] Differences in genotype and allele frequencies between study groups were estimated by chi-square test. The ORs were adjusted for confounding factors such as age and gender. In addition, the association between *MYBPC3* 25 bp deletion and significant risk factors of CAD were analyzed using binary logistic regression. Student t test was applied to find the significance for plasma cholesterol. Differences between groups stratified on the basis of genotypes were assessed using z proportional test. Overall we performed meta analysis by Stouffers method to combine the results of two cohorts (primary and replication) to calculate the overall z and p values. A two-tailed p-value of less than 0.05 was considered a statistical significant result. All statistical analyses were performed using SPSS software version 16.0 (SPSS, Chicago, IL, USA).
